# The Evolving Role of Bruton’s Tyrosine Kinase Inhibitors in B Cell Lymphomas

**DOI:** 10.3390/ijms25147516

**Published:** 2024-07-09

**Authors:** Shefali Mehra, Miah Nicholls, Justin Taylor

**Affiliations:** 1 Sylvester Comprehensive Cancer Center, University of Miami Miller School of Medicine, Miami, FL 33136, USA; shefalimehra@med.miami.edu; 2 College of Arts and Sciences, University of Miami, Coral Gables, FL 33146, USA; men67@miami.edu

**Keywords:** BTK, ibrutinib, B cell lymphomas, scaffolding function, PROTACs

## Abstract

Bruton’s tyrosine kinase (BTK), a non-receptor tyrosine kinase crucial for B cell development and function, acts downstream of the B cell receptor (BCR) in the BCR pathway. Other kinases involved downstream of the BCR besides BTK such as Syk, Lyn, PI3K, and Mitogen-activated protein (MAP) kinases also play roles in relaying signals from the BCR to provide pro-survival, activation, and proliferation cues. BTK signaling is implicated in various B-cell lymphomas such as mantle cell lymphoma, Waldenström Macroglobulinemia, follicular lymphoma, and diffuse large B cell lymphoma, leading to the development of transformative treatments like ibrutinib, the first-in-class covalent BTK inhibitor, and pirtobrutinib, the first-in-class noncovalent BTK inhibitor. However, kinase-deficient mutations C481F, C481Y, C481R, and L528W in the BTK gene confer resistance to both covalent and non-covalent BTK inhibitors, facilitating B cell survival and lymphomagenesis despite kinase inactivation. Further studies have revealed BTK’s non-catalytic scaffolding function, mediating the assembly and activation of proteins including Toll-like receptor 9 (TLR9), vascular cell adhesion protein 1 (VCAM-1), hematopoietic cell kinase (HCK), and integrin-linked kinase (ILK). This non-enzymatic role promotes cell survival and proliferation independently of kinase activity. Understanding BTK’s dual roles unveils opportunities for therapeutics targeting its scaffolding function, promising advancements in disrupting lymphomagenesis and refining B cell lymphoma treatments.

## 1. Introduction

Bruton’s tyrosine kinase (BTK) is a non-receptor tyrosine kinase representing a crucial signaling node in the B cell receptor (BCR) signaling pathway and plays a pivotal role in B-cell development, activation, and proliferation. BTK belongs to the Tec kinase family and serves as a linchpin in BCR signal transduction. Structurally, BTK comprises distinct domains, including an N-terminal pleckstrin homology (PH) domain crucial for membrane localization, a proline-rich Tec homology (TH) domain implicated in protein–protein interactions, an Src homology 3 (SH3) domain facilitating interactions with proline-rich sequences, an Src homology 2 (SH2) domain involved in substrate recognition, and a C-terminal kinase domain responsible for catalytic activity [1,2]. Through its intricate domain architecture, BTK translates extracellular stimuli received by the BCR into intracellular signaling events, thus regulating various immune cellular processes [3].

At the forefront of B cell signaling, the BCR pathway orchestrates a cascade of events upon antigen recognition, culminating in cellular responses essential for immune function. The BCR complex consists of membrane-bound immunoglobulin (Ig) molecules that recognize specific antigens, coupled with CD79A and B signaling subunits [4,5]. Upon antigen binding, the BCR complex undergoes receptor oligomerization and the activation of downstream signaling molecules [6].

Central to BCR signaling is the tyrosine kinase Lyn, a member of the Src family of kinases. Lyn is constitutively associated with the cytoplasmic tails of CD79A and CD79B subunits and phosphorylates immunoreceptor tyrosine-based activation motifs (ITAMs) within these subunits upon BCR engagement [7]. Phosphorylated ITAMs serve as docking sites for downstream signaling molecules, including spleen tyrosine kinase (Syk). Syk, a cytoplasmic tyrosine kinase, plays a role in transducing BCR signals upon binding to phosphorylated ITAMs. Structurally, Syk comprises two SH2 domains flanking a catalytic kinase domain. Upon recruitment to phosphorylated ITAMs, Syk undergoes autophosphorylation and activation, initiating downstream signaling cascades [8]. The activation of Syk leads to the phosphorylation and activation of downstream effectors, including phosphoinositide 3-kinase (PI3K) and MAP kinases [9]. PI3K, a heterodimeric enzyme comprising regulatory (p85) and catalytic (p110) subunits, catalyzes the conversion of phosphatidylinositol 4,5-bisphosphate (PIP2) to phosphatidylinositol 3,4,5-trisphosphate (PIP3), thereby activating downstream signaling pathways involved in cell survival, proliferation, and metabolism [5,10,11].

**Figure 1:**
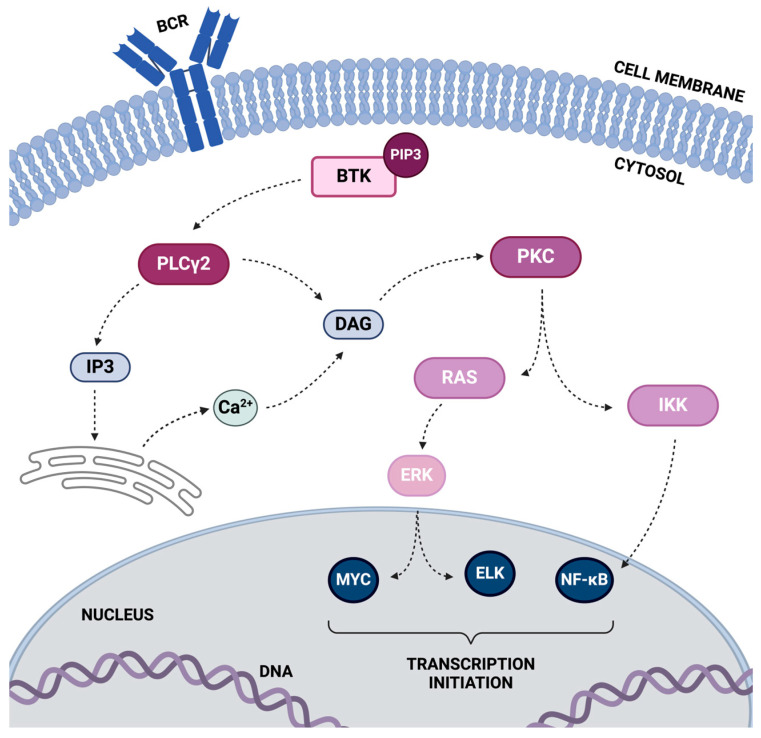
This schematic diagram depicts the signaling cascade downstream of Bruton’s tyrosine kinase (BTK) in the B-cell receptor (BCR) pathway, highlighting critical substrates that drive B cell proliferation, activation, survival, and differentiation. Upon BCR engagement, BTK phosphorylates phospholipase Cγ2 (PLCγ2), leading to the generation of inositol 1,4,5-trisphosphate (IP3) and DAG (diacylglycerol). IP3 mobilizes intracellular calcium, while DAG activates PKC (protein kinase c). This activation further stimulates the rat sarcoma (RAS) pathway, leading to the activation of extracellular signal-regulated kinase (ERK) and transcription factors (myelocytomatosis) MYC and ETS-like kinase (ELK). Concurrently, PKC activates the inhibitor of the nuclear factor-κB kinase (IKK) complex, resulting in the translocation of nuclear factor kappa-light-chain-enhancer of activated B cells (NF-κB) to the nucleus, promoting gene expression essential for B cell survival and proliferation.

Within the BCR pathway (Figure 1), BTK occupies a key position downstream of Syk. Upon activation by Syk, BTK undergoes conformational changes and translocation to the cell membrane facilitated by its PH domain. At the membrane, BTK becomes accessible to its substrate molecules and undergoes autophosphorylation at a conserved tyrosine residue within its kinase domain (Y551) [12]. Activated BTK then phosphorylates multiple downstream substrates, thereby propagating the BCR signaling cascade. One of the primary substrates of BTK is phospholipase Cγ2 (PLCγ2), which, upon phosphorylation, leads to its activation. Activated PLCγ2 cleaves PIP2 into inositol 1,4,5-trisphosphate (IP3) and diacylglycerol (DAG). IP3 triggers the release of calcium from intracellular stores, leading to calcium mobilization, while DAG activates protein kinase C (PKC), which in turn regulates various downstream signaling pathways involved in cellular responses [13].

In addition to PLCγ2, BTK activates several other downstream effectors, including MAP kinases such as extracellular signal-regulated kinases (ERKs), c-Jun N-terminal kinases (JNKs), and p38 MAP kinases. ERKs, a subgroup of MAPKs, are activated downstream of BTK [5,14]. Upon activation by BTK-mediated phosphorylation, ERKs translocate to the nucleus, where they phosphorylate transcription factors and modulate gene expression programs critical for B-cell activation and function [15]. Furthermore, BTK activation modulates the nuclear factor kappa-light-chain-enhancer of activated B cells (NF-κB) signaling pathway. The phosphorylation of specific NF-κB regulatory proteins downstream of BTK leads to the activation of NF-κB transcription factors, which translocate to the nucleus and regulate the expression of genes involved in immune responses, cell survival, and inflammation [16].

The interplay between the BTK pathway and other kinases, such as Lyn, Syk, PI3K, and MAP kinases, is integral to the regulation of B-cell signaling and function. Crosstalk between these pathways modulates the intensity and duration of BCR signaling, thereby fine-tuning cellular responses to antigen stimulation [13,17].

## 2. BTK and B Cell Lymphoma

Non-Hodgkin lymphomas (NHLs) represent a diverse array of lymphoid malignancies characterized by the clonal expansion of B or T lymphocytes. The aberrant activation of the BCR pathway stands prominently at the forefront of the pathogenesis of certain B cell NHL subtypes, facilitating uncontrolled B cell proliferation and survival. Dysregulated BCR signaling, often driven by mutations or overexpression of BCR components, engenders the sustained activation of BTK and its downstream effectors. BTK, expressed in all hematopoietic cells except for T cells, intricately regulates critical signaling events downstream of the BCR [17]. Mutations within the BCR complex, such as CD79A/B ITAM mutations, yield constitutive BTK activation, thereby fostering malignant transformation [18]. Kuo et al. were able to determine that a transcription factor, SOX11, was responsible for the hyperactivation of BTK, allowing for tumor development [19]. Moreover, the amplification of BTK expression has been notably observed in diffuse large B-cell lymphoma (DLBCL), correlating with disease aggressiveness and predicting poor prognosis [1,20].

Furthermore, the crosstalk between the BTK pathway and other signaling cascades significantly contributes to NHL pathogenesis. The activation of NF-κB and MAPK pathways downstream of BTK orchestrates anti-apoptotic gene expression and augments cell survival. Additionally, dysregulated PI3K/Akt signaling, frequently concomitant with BTK activation, fosters cell proliferation and drives metabolic reprogramming in NHL cells [1,21].

In summary, the dysregulated BCR pathway assumes a central role in certain NHL pathogenesis, fueling aberrant B-cell proliferation, survival, and migration [17]. Specific alterations in BTK’s regulatory elements, alongside perturbed BCR signaling, collectively contribute to sustained pathway activation and fuel tumor growth. A comprehensive understanding of the intricate mechanisms underpinning BCR-mediated lymphomagenesis not only elucidates the molecular landscape of NHL but also holds promise for the development of novel therapeutic strategies aimed at targeting this pathway for more effective B cell NHL treatment [6].

## 3. Targeting BTK in B Cell Lymphomas

The advent of BTK inhibitors ushered in a promising era in lymphoma therapeutics owing to the role played by BTK in the BCR pathway in the pathogenesis of lymphomas. Malignant B-cells exhibit a pronounced reliance on BTK activity for their survival, propelling the pursuit of BTK inhibitors as a targeted therapeutic strategy [2,22,23,24]. Subsequent efforts led to the development of BTK inhibitors such as PCI-32765, heralding a new era in lymphoma therapeutics. PCI-32765, now ibrutinib, emerged as a non-selective and irreversible small molecule inhibitor of BTK [25]. Its mechanism of action involved covalent bonding with the cysteine (Cys)-481 residue at the active site of BTK, resulting in the irreversible inhibition of tyrosine kinase activity at the tyrosine (Tyr)-223 position [24,26].

Following the preclinical validation of ibrutinib’s efficacy against malignant B cell proliferation by Honigberg et al., early Phase I trials commenced in 2006 for patients with chronic lymphocytic leukemia (CLL). Given the established link between constant B cell receptor (BCR) activation, mediated by BTK, and CLL pathogenesis, the hypothesis regarding the detrimental impact of BTK inhibition on lymphoma cell survival gained traction. In vitro experiments underscored ibrutinib’s ability to induce apoptosis in CLL cells via caspase activation, coupled with the inhibition of activation-dependent cell proliferation [27].

The Phase I trial, encompassing 47 patients with CLL and mantle cell lymphoma (MCL), yielded encouraging results, with three patients achieving complete responses (CRs) and seventeen demonstrating partial responses (PRs). Notably, a significant proportion of patients maintained trial participation for over six months, notwithstanding manageable Grade 3 toxicities, predominantly Grade 3 neutropenia observed in nine patients (19%) [26,28].

Bolstered by favorable responses observed in MCL patients, clinical trials expanded in 2012 to encompass a spectrum of relapsed and refractory non-Hodgkin lymphomas (NHLs) [29,30]. Advani et al.’s Phase I trial, inclusive of patients with various B cell malignancies, yielded promising outcomes, with 60% of patients achieving either CR or PR, across diverse histologies, including mantle cell, mucosal associated lymphoid tissue, follicular, and DLBCL. Additionally, responses were recorded across all histologies with CRs being seen for patients with MCL (3/9 patients) and follicular lymphoma (3/16 patients) [31].

The milestone FDA approval of ibrutinib for the treatment of relapsed MCL in 2013 heralded a paradigm shift in lymphoma management [10]. While this approval extended to include treatment for CLL and Waldenström Macroglobulinemia (WM) during the next couple of years, the adverse effects of using a non-selective inhibitor became apparent in many of these clinical trials. While ibrutinib was primarily responsible for targeting BTK, it also inhibited several other kinases, including (Interleukin-2-Inducible T-Cell Kinase) ITK, (Tyrosine Kinase Expressed in Hepatocellular Carcinoma) TEC, and (Tyrosine Kinase Non-Receptor 1) TXK [32]. The inhibition of these kinases resulted in off-target side effects and a higher incidence of adverse events. In trials using ibrutinib as treatment for patients with CLL, MCL, and MCL, up to 16% of patients developed atrial fibrillation as a result. Additionally, patients treated with ibrutinib for CLL, MCL, or WM were found to have a significant 4.85% increased all-grade bleeding risk when compared to control groups, which had 1.1% increased all-grade bleeding risk [33,34].

Coupled with an increased risk of adverse effects and decreased specificity and targeted inhibition of BTK, the foundation for the development of the next generation of BTK inhibitors was laid. Subsequent approvals of a second generation of BTK inhibitors such as acalabrutinib (formerly ACP-196), orelabrutinib (ICP-022), and zanubrutinib (BGB-3111) for lymphoma treatment soon followed. These second-generation BTK inhibitors all consisted of altered chemical structures allowing for the increased targeting of the BTK enzyme, when compared to the non-selective ibrutinib. Acalabrutinib, while also being able to covalently bond to the Cys-481 residue like ibrutinib, was able to show greater selectivity for BTK when compared to ibrutinib and has also been approved to treat patients with MCL and CLL. Zanubrutinib, containing a purine core and acrylamide moiety that binds covalently to the cysteine residue (Cys-481) in the BTK enzyme similar to acalabrutinib, has an additional pyrazolo[3,4-d] pyrimidine scaffold which facilitates heightened BTK selectivity. Zanubrutinib has received approval for the treatment of MCL and CLL and is currently being used in clinical trials for patients with WM and DLBCL [35,36,37]. The structure of orelabrutinib consists of a distinct scaffold with a urea linkage, allowing it to interact with the BTK active site specifically, and its structure allows for decreased interaction and selectivity for other kinases, thus preventing off-target binding [38].

The results for patients treated with second-generation BTK inhibitors have been promising. Zanubrutinib has shown impressive results in various studies, particularly for relapsed/refractory B-cell lymphomas. In the phase 3 ALPINE trial, zanubrutinib demonstrated superior progression-free survival (PFS) compared to ibrutinib in patients with relapsed/refractory CLL and SLL [39]. Specifically, zanubrutinib reduced the risk of disease progression by 35% and had a 24-month PFS rate of 78.4% compared to 65.9% for ibrutinib. Additionally, zanubrutinib showed a better cardiac safety profile with fewer serious cardiac adverse events compared to ibrutinib [40]. Acalabrutinib has also shown significant efficacy in treating B-cell lymphomas. In the phase 3 ELEVATE-TN trial, acalabrutinib was compared to standard chemoimmunotherapy in previously untreated CLL. Acalabrutinib, either alone or in combination with obinutuzumab, significantly improved PFS compared to chemoimmunotherapy. The study showed that acalabrutinib alone reduced the risk of disease progression or death by 69% compared to chemoimmunotherapy [41]. Finally, in a phase 2 study, patients with relapsed/refractory MCL being treated with orelabrutinib demonstrated a high overall response rate (ORR) of 83% [42].

## 4. Development of BTK Inhibitor Resistance

Despite these advancements, resistance and disease progression emerged as formidable challenges among patients receiving covalent BTK inhibitors [43,44]. Notably, patients with MCL exhibited therapy failure with ibrutinib, and relapsed patients experienced dismal outcomes, typified by a median overall survival of merely 8.4 months post-ibrutinib discontinuation (prior to the development of the BCL-2 inhibitor venetoclax). Furthermore, MCL patients treated with second-generation BTK inhibitors such as acalabrutinib and zanubrutinib also developed resistance to the therapeutics [45]. This led to the development of non-covalent reversible BTK inhibitors (ncBTKis) to address concerns of covalent BTK inhibitor resistance and toxicities. By reversibly binding to BTK via hydrogen or ionic bonding as well as hydrophobic interactions, non-covalent BTK inhibitors do not require C481 in order to elicit their effects [46]. Vecabrutinib was one of the first non-covalent BTK inhibitors developed; however, the CLL program was halted due to a lack of efficacy. Pirtobrutinib and nematabrutinib are orally available, and reversible noncovalent BTK inhibitors that were next to be studied, with pirtobrutinib now gaining approval for relapsed, refractory MCL and CLL. In the BRUIN study, which included CLL, MCL, DLBCL, WM, FL, marginal zone lymphoma (MZL), and primary central nervous system lymphoma (PCNSL), patients treated with pirtobrutinib, formerly LOXO-305, and patients with heavily relapsed and refractory CLL, including those previously treated with BTK inhibitors, were shown to have an overall response rate of 74% [47].

**Figure 2:**
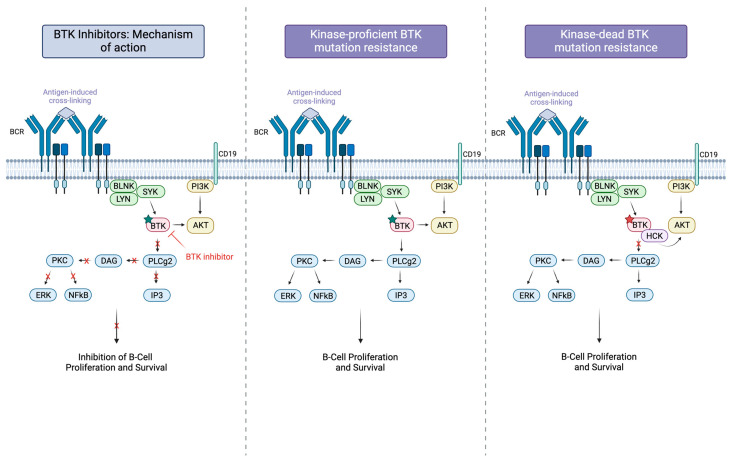
This schematic diagram illustrates the mechanisms of action and resistance to Bruton’s tyrosine kinase (BTK) inhibitors in B cell receptor (BCR) signaling. The left panel shows BTK inhibitors blocking BTK activity, resulting in the inhibition of downstream signaling pathways, including PKC, DAG, PLCγ2, IP3, ERK, and NF-κB, thereby inhibiting B cell proliferation and survival. The middle panel represents kinase-proficient BTK mutation resistance, where mutations allow BTK to remain active despite inhibitor presence, maintaining BCR signaling and B cell proliferation. The right panel depicts kinase-impaired BTK mutation resistance, where alternative kinases like HCK compensate for impaired BTK activity, sustaining downstream signaling and B cell proliferation. Green stars indicate active BTK and red stars indicate kinase impaired BTK.

Despite initial success with treating ibrutinib-resistant lymphomas with non-covalent BTK inhibitors, soon thereafter, studies demonstrated patients experiencing resistance to this new line of therapeutics. While C481 mutations were overcome with non-covalent BTK inhibitors, the discovery of other sites of mutation provided an explanation for resistance in these patients. Mutations in BTK such as C481S and T474I prevent drugs from binding (covalent for C481S and both covalent and non-covalent for T474I) yet allow for continued BTK enzymatic activity. Particular mutations in BTK at C481F, C481Y, C481R, V416L, A428, and L528W have been shown to render the protein kinase-deficient in malignant cells in vitro, hence resulting in inherent resistance to drugs targeting the kinase activity of BTK (Figure 2) [48]. Of note, 80% of patients who experience ibrutinib therapy failure have been found to have PLCG2 gene mutations in addition to BTK gene mutations. These mutations result in heightened sensitivity to phosphorylation by BTK and Syk, consequently amplifying the activation of PLCγ2 [49,50].

Other mechanisms of resistance to BTK inhibitors pose a significant challenge in the treatment of NHLs, such as WM. Interestingly, WM patients, who demonstrate *MYD88* mutations, were shown to have a 95% response rate to ibrutinib in a study conducted by Castillo et al. [51]. While the reasons for decreased *BTK* gene mutation mediating resistance to BTK inhibitors for patients with WM remain relatively unexplored, it is thought that *CXCR4* mutations may confer resistance to BTK inhibitors instead. Similarly, primary resistance to ibrutinib in MCL often stems from the chronic activation of the MAP3K14-NF-κB pathway, leading to constitutive NF-κB activation. Additionally, mutations in NF-κB inhibitors (TRAF2, TRAF3, BIRC3) contribute to primary resistance, with loss-of-function mutations, particularly in BIRC3, resulting in the activation of non-canonical NF-κB signaling pathways, conferring resistance [52,53].

Furthermore, mutations in *CARD11* downstream of BTK and the regulation of NF-κB activity are also linked to primary resistance to ibrutinib. Chromosomal abnormalities such as chromosome 9p loss containing the SMARCA2 genomic region, deletions in ARID2, or mutations in SMARCA4 have been associated with resistance to ibrutinib [54]. Genetic alterations in chromosomes 9p, 6q, and 13q are commonly observed in ibrutinib-resistant MCL tumors, suggesting their involvement in mediating resistance mechanisms [55].

In addition to genetic factors, non-genetic causes contribute to ibrutinib resistance. Intrinsically, ibrutinib-resistant DLBCL cell lines often exhibit the overexpression of CD79B and the activation of the Akt/MAPK pathway, leading to resistance. MYC overexpression has been identified as an intrinsic cause of ibrutinib resistance in MCL. Furthermore, the activation of non-canonical NF-κB and MAPK pathways through CD40L-CD40 signaling can bypass BTK signaling, diminishing ibrutinib efficacy in MCL. Targeting exportin-1 (XPO1) with selective inhibitors sensitizes intrinsic ibrutinib-resistant MCL cell lines by inhibiting NF-κB signaling [56,57].

The tumor microenvironment (TME) plays a crucial role in ibrutinib resistance, with stromal cells like mesenchymal stromal cells (MSCs) and myeloid cells promoting tumor growth and resistance. Interaction with MSCs activates the PI3K-mTOR pathway and integrin-β1 signaling, contributing to resistance. The ibrutinib-mediated modulation of the TME involves disrupting oncogenic signaling axes and inhibiting growth-supportive cytokines and growth factors, highlighting the dynamic interplay between the TME and tumor cells in driving resistance mechanisms to BTK inhibitors [50,58].

## 5. Noncatalytic Scaffolding Function of BTK

The discovery of non-enzymatic functions of BTK began with seminal studies aimed at elucidating the molecular mechanisms underlying X-linked agammaglobulinemia (XLA), a primary immunodeficiency disorder resulting from mutations in the BTK gene. The function of BTK in B cell development independent of its catalytic activity was first outlined by Middendorp et al. by using mice expressing a mutant Btk with a tyrosine to phenylalanine mutation at position 223 (Y223F-Btk) and mice expressing a kinase-inactive mutant Btk with a lysine to arginine mutation at position 430 (K430R-Btk). They were able to determine that Y223 autophosphorylation was not needed for peripheral B cell differentiation and changes in pre B cells; alternatively, kinase-inactive B cells were able to reconstitute B cell activity in BTK-deficient mice as well as partially correct the defective modulation of pre-B cell surface markers, peripheral B cell survival and BCR-mediated NF-kB induction [59].

The discovery of the non-enzymatic function of BTK marked a significant milestone in understanding the multifaceted roles of this protein beyond its classical enzymatic activity that allows for the propagation of malignancy. Yuan et al. determined that kinase-inactive forms of BTK in DLBCL cells such as C481F, C481Y, C481R, and L528W maintained BCR signaling [60]. By recreating these unique mutations using CRISPR/Cas9 gene editing in BTK along with transduction into DLBCL cells, Yuan et al. targeted several key genes involved in BCR signaling pathways using signal-guide RNAs (sgRNAs). These genes included CD79A/CD79B, SYK, BLNK, PLCG2, PRKCB, CARD11/BCL10/MALT1, and NFKB1. DLBCL cells harboring BTK-inactivating mutations remained dependent on these BCR signaling pathway genes for continued survival [61].

Additionally, Yuan et al. found that Toll-like receptor 9 (TLR9), UNC93B1, and CNPY3 formed a protein–protein interaction network in the L528W knock-in DLBCL cells. TLR9 is known for being involved in sensing microbial DNA and triggering proinflammatory signaling, while UNC93B1 and CNPY3 are involved in proper TLR9 folding and localization to endosomes, respectively [62,63]. They observed the depletion of sgRNAs targeting the nonreceptor tyrosine kinase HCK in L528W knock-in cells. By using a competitive cell growth assay, cells with kinase-inactive BTK (C481F/Y/R and L528W) were depleted more rapidly when transduced with sgRNAs targeting TLR9, UNC93B1, CNPY3, or HCK compared to cells with kinase-active BTK (C481S).

Transitioning from the discovery of BTK’s non-enzymatic functions to the elucidation of its scaffolding function represented a paradigm shift in the understanding of its molecular mechanisms of action [64]. Initially characterized as a canonical signaling kinase orchestrating phosphorylation-dependent signaling cascades, BTK was later found to act as a molecular scaffold, facilitating protein–protein interactions and the spatial organization of signaling complexes within the cell [65].

In particular, BTK has been shown to activate integrins downstream of the BCR. Spaargaren et al. demonstrated that BTK activation was necessary for the integrin alpha-4-beta-1 (VLA-4)-mediated adhesion of B cells to vascular cell adhesion molecule −1 and fibronectin. Additionally, BTK was found to play a role in the control of the integrin-mediated adhesion of pre-B cells. This study revealed new roles for BCR and BTK with regard to B cell development and differentiation. The interaction of integrin alpha-4-beta-1 and fibronectin was found to be responsible for controlling early B cell development into pro-, pre-, and immature B cells, which would then play a key role in antigen-specific B cell differentiation. During antigen-specific B cell differentiation, after the formation of germinal centers, wherein B cells would undergo expansion, the representation of antigen by follicular dendritic cells (FDCs) would allow for B cells expressing high affinity BCR for antigen and undergo isotype switching with further maturation into plasma or memory B cells. One of the integrins which mediated the interactions between FDCs and high affinity BCR was found to be alpha-4-beta-1, which engages with vascular cell adhesion protein 1 (VCAM-1) on the FDC. In DT40 cells which were BTK-deficient, Spaargen et al. observed a complete loss of the BCR-mediated adhesion of B cells to VCAM. While these findings provided crucial insights into the canonical enzymatic role of BTK in phosphorylation-mediated signaling pathways, they also hinted at additional functions that remained to be explored [66].

One of the seminal findings that underscored the non-enzymatic functions of BTK was its association with actin cytoskeleton remodeling. Not only did BTK have non-enzymatic capabilities with integrin control but also it plays a role in actin skeleton reorganization in order to facilitate the efficient transport and presentation of antigens by B cells [67]. By using a combination of BTK-deficient mice cells and a BTK inhibitor, Sharma et al. demonstrated that actin polymerization was dependent on BTK activity. They observed increased levels of phosphatidyllinositide-4,5-bisphosphonate and phosphorylated Vav, molecules which aid actin cytoskeleton reorganization, upon BCR engagement in a BTK-dependent manner. Furthermore, B cells treated with BTK inhibitors were shown to have late movement to endosomes and delayed BCR internalization. Therefore, through its interaction with actin-binding proteins such as filamin and Nck, BTK was found to modulate cytoskeletal rearrangements critical for cellular processes such as cell adhesion, migration, and phagocytosis [68].

The interactions of BTK with integrins and actin not only highlighted the multifunctional nature of BTK but also demonstrated its involvement in the regulation of fundamental cellular functions independent of its kinase activity. Roman-Garcia et al. detailed the role BTK played in the formation of an antigen-triggered immune synapse (IS formation). Cells with a mutation at the BTK PH domain, preventing BTK transport to the plasma membrane, were observed to have a decreased ability to form IS. It was also determined in vitro that B cells with impaired scaffolding functions would form a peripheral supramolecular activation cluster (pSMAC), the integrin- and F-actin-rich peripheral domain that has an integral role in the stabilization of the IS. Most importantly, they were able to show that a deficiency in the membrane recruitment of BTK was detrimental to the antigen-triggered microtubule-organization center polarization to the IS. Without polarization, in the presence of antigens, B cells with non-catalytic BTK were unable to proliferate [69].

Finally, further investigations looking into the scaffolding function of BTK includes studies conducted by Montoya et al. where researchers performed the immunoprecipitation of BTK from TMD8 cells overexpressing either wild-type BTK (BTK WT) or the kinase-impaired BTK L528W mutant tagged with a 3X FLAG tag [70]. They then analyzed the interactome of BTK WT and BTK L528W using mass spectrometry to identify proteins uniquely interacting with the mutant BTK L528W. To validate their mass spectrometry findings, the authors performed two-dimensional differential gel electrophoresis (2D-DIGE) analyses followed by the quantitative protein identification of proteins immunoprecipitated from BTK in the knock-in TMD8 cell lines. This orthogonal method confirmed differential protein interactions between wild-type and mutant BTK. The IP/mass spectrometry experiments revealed distinct interactions of BTK L528W with hematopoietic cell kinase (HCK) and integrin-linked kinase (ILK). HCK is a member of the SRC-family tyrosine kinases known to play a role in hematologic malignancies. ILK is a serine/threonine kinase upregulated in malignant cells, implicated in tumorigenesis, cancer cell proliferation, and drug resistance [71]. Ultimately, BTK L528W mutant cells were found to be preferentially dependent on HCK or ILK for cell proliferation and BCR signaling compared with BTK WT cells. BTK mutants resistant to BTK inhibitors achieved this effect by enhancing physical interactions with signaling molecules such as HCK and ILK, bypassing the requirement for BTK phosphorylation at tyrosine 223 to sustain downstream BCR signaling. These findings shed light on a novel mechanism by which BTK mutants confer resistance to BTK inhibitors and sustain downstream BCR signaling through enhanced interactions with signaling molecules such as HCK and ILK [70].

## 6. Targeting Noncatalytic Functions of BTK

The discovery of new functions of BTK revealed a need for new treatment avenues for patients with BTK-dependent lymphomas. Targeting enzyme activity meant that only one aspect of BTK activity would be targeted, which is likely insufficient. Hence, the importance of targeting all aspects of BTK function may help circumvent resistance.

Currently, proteolysis-targeting chimeras (PROTACs) have been used to degrade BTK and target the noncatalytic activity of BTK in malignant cells (Table 1). PROTACs are specialized molecules consisting of three parts, a component that binds to the protein of interest (POI), a linker, and a section that binds to E3 ubiquitin ligase [72,73]. When the PROTAC molecule interacts with both the E3 ligase and the target protein, it forms a complex called POI-PROTAC-E3 ligase. This complex manipulates the ubiquitin–protease system (UPS), leading to the polyubiquitination of the target protein. Subsequently, the protein undergoes proteasomal degradation [72,73,74].

NX-2127 is at the forefront of novel BTK PROTAC therapeutics being developed, which consists of a heterobifunctional molecule that engages the intracellular ubiquitin–proteasome system to simultaneously bind both BTK and the CRBN E3 ubiquitin ligase complex. This induces polyubiquitination and the subsequent proteasome-dependent degradation of BTK, as well as IKZF1 and IKZF3 proteins. Unlike traditional small-molecule inhibitors, protein degraders harness the cell’s protein degradation machinery to eliminate target proteins, offering advantages in selectivity and prolonged pharmacological effects. IKZF1 (Ikaros) and IKZF3 (Aiolos) are key lymphocyte transcription factors involved in the regulation of cytokines and the proliferation and activation of B cells, T cells, and natural killer cells [74].

The degradation of these proteins by NX-2127 has been shown to have therapeutic implications in various B cell malignancies. A total of 23 CLL/SLL patients were enrolled in the Phase I clinical trial (NCT04830137) across various dose levels. Before treatment with NX-2127, baseline blood specimens from 21 CLL patients were assessed for BTK mutational status. The sequencing analysis revealed a diverse range of mutations, including kinase-impaired mutations (L528W, C481R, and/or V416L) and kinase-proficient mutations (C481S, T474F, and/or T474I). Serial intracellular flow cytometric analysis of BTK degradation in peripheral blood B cells (CD19+CD3−) was performed to assess the kinetics of BTK degradation in patients treated with NX-2127. BTK degradation was quantified using BTK mean fluorescence intensity (MFI) measurements. NX-2127 exposure resulted in BTK degradation in circulating B cells, with maximal degradation observed as early as day 8 of the first cycle. The rate and degree of degradation were similar across all patients, regardless of their mutational status. Furthermore, sustained BTK degradation was observed throughout the daily dosing interval, independent of the mutation type and level of enzymatic activity. The clinical activity of NX-2127 was observed in CLL patients, including those with BTK resistance mutations acquired as a result of prior BTK inhibitor therapies. An improvement in the sum of the product of the diameters (SPD) of target lesions was observed in the majority of evaluable CLL patients (11 out of 14). This improvement was seen in patients harboring both kinase-proficient and kinase-impaired BTK resistance mutations, as well as various other recurrent CLL mutations [70].

Another PROTAC being investigated in adults with relapsed/refractory B cell malignancies is NX-5948, which selectively degrades BTK and not IKZF1/3 (NCT05131022). Currently, seven patients have been enrolled in phase 1a, receiving doses of 50 mg or 100 mg. Preliminary results indicate that NX-5948 exhibits dose-proportional pharmacokinetics, with a half-life of approximately 12 h and rapid, robust, and sustained degradation of BTK observed in all patients [72,75].

Another PROTAC for BTK is being tested in the BGB-16673-101 trial, an open-label, phase 1 study (NCT05006716) evaluating the safety, tolerability, and efficacy of BGB-16673 in patients with relapsed/refractory B cell malignancies, including CLL, WM, MCL, MZL, non-germinal center B-cell DLBCL, FL, and RT. Patients must have received at least two prior therapies (except for RT) and have an ECOG performance status of 0–2. The study utilizes dose escalation with six planned dose levels (50–600 mg once daily) using a Bayesian optimal interval design. Primary objectives include assessing safety, establishing the maximum tolerated dose (MTD), and determining the recommended phase 2 dose. Secondary objectives involve evaluating pharmacokinetics, pharmacodynamics, and preliminary antitumor activity. As of 26 May 2023, 26 patients have been enrolled across five dose levels. The median age was 70.5 years, and the median number of prior therapies was 3.5. Most patients had received prior covalent BTK inhibitors. Treatment-emergent adverse events (TEAEs) were common, with the most frequent being contusion, pyrexia, neutropenia, and increased lipase levels. No dose-limiting toxicities were observed, and the MTD was not reached. BGB-16673 exposure increased with dose, and preliminary pharmacodynamic data showed significant reductions in BTK protein levels in both peripheral blood and tumor tissue. Most CLL patients experienced lymphocytosis during treatment. Of the response-evaluable patients, 67% responded to treatment, including patients previously treated with covalent or non-covalent BTK inhibitors. The responses were durable, with the longest responder remaining on treatment for 60 weeks [76].

## 7. Conclusions and Future Directions

The B cell receptor (BCR) signaling pathway and its central mediator, Bruton’s tyrosine kinase (BTK), regulate immune responses in normal hematopoietic cells but can contribute to lymphomagenesis. The activation of BTK via the BCR causes signal transduction to mediators of cellular processes critical for B cell function and survival. The pivotal role of BTK in BCR signaling and B cell lymphoma pathogenesis has led to the development of BTK inhibitors as targeted therapies, which are now a frontline therapy for many lymphoma subtypes. Ibrutinib, the first FDA-approved BTK inhibitor, has demonstrated remarkable efficacy in treating relapsed/refractory MCL, CLL, and other B-cell malignancies. Subsequent generations of BTK inhibitors, such as acalabrutinib and zanubrutinib, offer enhanced selectivity and efficacy, expanding treatment options for patients. Despite the development of non-covalent BTK inhibitors, such as pirtobrutinib, in order to overcome C481 BTK resistance mutations seen after covalent BTK inhibition, newly discovered kinase-deficient BTK resistance mutations seen after covalent and noncovalent BTK inhibitor treatment prompted further investigations into BTK’s scaffold function.

The emergence of resistance to covalent and non-covalent BTK inhibitors poses a significant clinical challenge, necessitating a deeper understanding of resistance mechanisms and the development of novel therapeutic strategies. Efforts to target the noncatalytic scaffolding functions of BTK, alongside its enzymatic activity, offer promising avenues for overcoming resistance and improving patient outcomes. Innovative approaches, including protein degraders like PROTACs, such as NX-2127, NX-5948, and BGB-16673, represent a novel approach to target BTK and IKZF1/3 proteins, offering potential benefits in the treatment of B cell malignancies, including those resistant to traditional small-molecule inhibitors. When comparing PROTACS to BTK inhibitors, there are also different pharmacokinetic stoichiometries, with one PROTAC molecule being able to degrade multiple BTK molecules. Ultimately, the choice between these molecules will be heavily influenced by clinical trial outcomes, including efficacy, safety profiles, and any observed resistance mechanisms with the PROTACs.

By targeting the multifaceted functions of BTK and its interplay with the tumor microenvironment, researchers strive to optimize therapeutic strategies and improve outcomes for patients with B-cell malignancies. While there is an inherent need for trials to expand and include patients with other types of B cell lymphomas, the discovery of additional scaffolding functions for BTK in addition to novel therapeutics targeting these functions will offer new options for clinicians treating patients with malignancies relapsed and refractory to current BTK inhibitors.

## Figures and Tables

**Table 1:** Table listing clinical trials of current Bruton’s tyrosine kinase (BTK) proteolysis-targeting chimeras (PROTACs), including clinicaltrials.gov number, study arms, and criterion for prior therapy for administration of PROTAC in each arm.

Clinical Trial Number/Intervention [Phase]	Study Arms	Prior Therapy *
NCT05131022/NX-5948 [Phase I]	CLL/SLL	BTKi + BCL-2 inhibitor
	MCL	BTKi + anti-CD20 mAb-based regimen
	WM	BTKi + additional line of therapy
	DLBCL	Anthracycline (unless ineligible) + anti-CD20 mAb-based regimen + additional line of therapy
	FL (grade 1-3a)	Anti-CD20 mAb-based regimen + additional line of therapy
	PCNSL	2 lines of therapy
	MZL	Anti-CD20 mAb-based regimen + additional line of therapy
NCT04830137/NX-2127 [Phase I]	CLL/SLL (no BTK C481 mutation)	BTKi
	CLL/SLL (BTK C481 mutation present)	BTKi
	MCL	BTKi + anti-CD20 mAb-based regimen
	DLBCL; WM	Anti-CD20 mAb-based regimen + anthracycline/anti-CD 19-based regimen/other palliative; BTKi
	FL/MZL; PCNSL	Anti-CD20 mAb-based regimen; 1 line of treatment
NCT05006716/BGB-16673 [Phase II]	MCL	BTKi
	CLL/SLL	BTKi + BCL-2 inhibitor
	MZL, FL, WM, DLBCL, >2 treatments per the Richter’s transformation to DLBCL	±BTKi

* Patients must have failed/disease progression/significant side effects with prior therapy. CLL (Chronic Lymphocytic Leukemia), SLL (Small Lymphocytic Lymphoma), BTKi (Bruton Tyrosine Kinase inhibitor), BCL-2 (B-Cell leukemia/lymphoma Receptor-2), MCL (Mantle Cell Lymphoma), WM (Wäldentsröm Macroglobulinemia), DLBCL (Diffuse Large B-Cell Lymphoma), FL (Follicular Lymphoma), PCNSL (Primary Central Nervous System Lymphoma), MZL (Marginal Zone Lymphoma), mAb (monoclonal antibody).
